# Effect of Microwave Treatment on Adzuki Beans (*Vigna angularis L.*) under Dry State—Analyzing Microstructure, Water Absorption, and Antioxidant Properties

**DOI:** 10.3390/foods11111653

**Published:** 2022-06-03

**Authors:** Seon-Min Oh, Seok-Bo Song, Jeom-Sig Lee, You-Geun Oh, Yu-Chan Choi, Jeong-Heui Lee, Jieun Kwak

**Affiliations:** 1 Department of Central Area Crop Science, National Institute of Crop Science, Rural Development of Administration (RDA), Suwon 16613, Korea; seonminoh@korea.kr (S.-M.O.); leejsyr@korea.kr (J.-S.L.); oyg0723@korea.kr (Y.-G.O.); oksusu1@korea.kr (Y.-C.C.); lejehe@korea.kr (J.-H.L.); 2 Department of Southern Area Crop Science, National Institute of Crop Science, Rural Development Administration, Miryang 50424, Korea; songsb1254@korea.kr

**Keywords:** adzuki bean, microwave treatment, water absorption behavior, softening, antioxidant activity

## Abstract

In this study, a microwave was used on adzuki beans (Arari and Geomguseul) without water, in order to investigate their changes in microstructure, water absorption, and antioxidative properties. As the microwave treatment time increased (2450 MHz, 0 to 60 s), the lightness, redness, and yellowness were reduced, and moisture content significantly decreased in both varieties. The microstructure space between the seed coat, cotyledon, and pores within the cotyledon were observed, due to the loss of moisture. Regardless of microwave treatment, the water absorption behavior of the adzuki beans was sigmoidal. However, the water absorption kinetics of Arari increased after microwave treatment, whereas with microwave-treated Geomguseul, the water absorption rate decreased, compared to the control, except for the sample treated for 30 s. During soaking, the water absorption and softening rates in the microwave-treated adzuki bean were twice as fast as the untreated beans. Antioxidant activity, total phenolic compounds, and total flavonoid compounds were greatly improved by microwave treatment. These results indicate that microwave treatment affects the color, hydration, and bioactive compounds, and it can be used as a pretreatment method before processing adzuki beans.

## 1. Introduction

The adzuki bean (*Vigna angularis*) is a crop belonging to the Fabaceae family, and it is mainly cultivated and used in East Asian countries, such as Korea, Japan, and China. Adzuki bean has been used as an herbal medicine since ancient times, and it is mainly used for cooking with rice or as a material for porridge, pastes, and dessert [1,2]. Generally, red-colored adzuki beans are distributed in the market; however, adzuki beans of various colors are used (black, yellow, and green), according to the purpose. Although some differences exist, depending on the variety, adzuki beans are regarded as an excellent source of nutrition because they have a protein content of over 20% and carbohydrate content of over 60% [3,4]. In addition, adzuki beans have many bioactive compounds that have attracted attention because of their considerable health benefits and functionality. Durak et al. [5] investigated the antihypertensive properties and antiradical activity of peptides in the adzuki bean protein fraction and suggested that adzuki beans are beneficial for human health. Experiments conducted on animals indicated that adzuki bean extracts improve lipid metabolism in both normal and high-fat diet groups [6], as well as that flavonoids and saponins present in adzuki beans contribute to the inhibition of α-glucosidase and pancreatic lipase activities [7].

As the health benefits of adzuki bean are commonly known, the demand for adzuki beans is increasing in the market, and various adzuki bean products are being developed. Before processing for consumption, adzuki beans must first be soaked in water to increase the weight of the dry seeds two-fold, which typically requires more than 20 h, and then cooked [3,8,9,10]. Hydration is an important process related to cooking quality; however, soaking for such a long duration is inconvenient for consumer and industrial purposes; hence, this problem should be improved by inducing a change of the microstructure. Thereafter, upon cooking (such as boiling and baking) the soaked adzuki bean, volatile compounds having a pleasant flavor are formed, but some functional compounds were lost. Aguilera et al. [11] reported that soaking and cooking decreased the flavonol and flavanone levels of soybeans. When the adzuki bean with water was autoclaved, a higher amount of functional compounds was released, compared to the untreated adzuki bean [12]. Therefore, pretreatment that contributes to structural changes and functional improvement simultaneously is required in the processing of adzuki beans. However, pretreatment of adzuki beans in wet conditions may make them vulnerable to microorganisms and reduce their shelf life. Furthermore, the extra drying process before distribution leads to defects in the quality of the adzuki bean, not only low economic effectiveness. Therefore, dry heating treatment is considered to be a suitable pretreatment method for adzuki beans.

Microwaves are electromagnetic waves with frequencies of 300–300,000 MHz, and they generate heat by intermolecular friction as the dipoles of the molecules in the food absorb energy [13]. Microwaves are applied in various processes, such as drying, sterilization, and thawing. They present the advantages of reduced cooking time and facilitated operation, due to their fast heating rate, which affects the structure and characteristics of food materials during processing. It is reported that, as the microwave treatment proceeds, bulk density reduces, surface area increases [14], and heat mass transfer accelerates, resulting in reduced cooking time [15]. During microwave treatment, phenolic polymerization and Maillard reaction contribute to the formation of colored compounds, called melanoidins, which provide a dark color to roasted foods [16]. Considering the effect of microwave treatment on other legumes, it is expected to change the characteristics of adzuki beans.

To the best of our knowledge, there is little information regarding microwave-treated adzuki bean seeds without water addition. Therefore, the effect of microwave pretreatment on processing-related quality characteristics, namely microstructure, water absorption, and antioxidative properties, was investigated, in order to discuss the possibility of its application as an adzuki bean processing technology.

## 2. Materials and Methods

### 2.1. Materials

**Figure 1:**
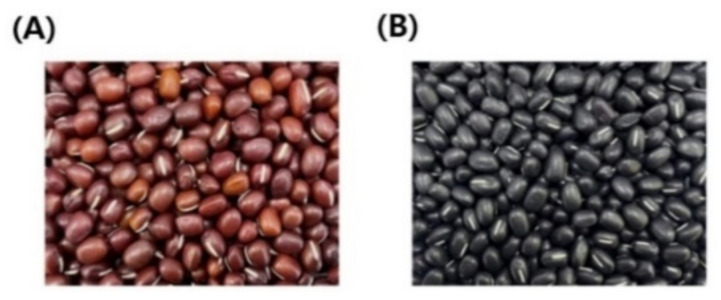
Images of two adzuki bean varieties: Arari (**A**) and Geomguseul (**B**).

The Korean adzuki bean varieties used in this study were Arari and Geomguseul, cultivated in Miryang (Gyeongsangbuk-do, Korea), and had red- and black-colored seed coats, respectively (Figure 1). Adzuki beans were stored at 4 °C and allowed to stand for 1 h at room temperature (25 °C) before use. All reagents used in this study were of analytical grade.

### 2.2. Microwave Treatment

Adzuki beans (30 g) were poured into a porcelain mortar and treated using a microwave oven (RE-IH700N, Samsung, Seoul, Korea) with 700 W output and a frequency of 2450 MHz for 30 s, 60 s, and two sets of 30 s at intervals of 1 min. All microwave treatments were carried out in the absence of water, and they were replicated three times. The surface temperature of the treated adzuki beans was measured using a thermometer (GS320, Inparo Co., Suwon, Korea) immediately after each respective microwave treatment: Arari: 119.1 ± 2.1 °C (30 s), 138.9 ± 3.9 °C (30–30 s), and 141.7 ± 3.52 °C (60 s); Geomguseul: 113.2 ± 3.68 °C; 30 s), 138.5 ± 2.7 °C (30/30 s), and 139.6 ± 2.8 °C (60 s). Treated adzuki beans were cooled to room temperature for 30 min and stored in a desiccator. Untreated adzuki beans were used as controls.

### 2.3. Color Analysis

The untreated and microwave-treated adzuki beans were filled into 10 mm thick plastic cells (M-A131, Minolta, Tokyo, Japan) and analyzed using a colorimeter (CM-3500d, Minolta, Tokyo, Japan) at 25 °C. The L* (lightness), a* (redness), and b* (yellowness) coordinates ranged from L = 0 (black) to 100 (white), a = −80 (green) to 100 (red), and b = −80 (blue) to 70 (yellow). The standard values for the white calibration plate were L = 98.8, a = −0.09, and b = −0.37. The color differences (ΔE) between the control and samples were calculated using Equation (1):

(1) $$\Delta E=\sqrt{{\left(\Delta L\right)}^{2}+{\left(\Delta a\right)}^{2}+{\left(\Delta b\right)}^{2}}$$

### 2.4. Scanning Electron Microscope

The microstructures of the control and microwave-treated adzuki beans were observed using a scanning electron microscope (TM3000, Hitachi, Tokyo, Japan). The samples were cut horizontally, and the cut cross-sections were observed. All specimens were observed at an accelerating voltage of 15 kV.

### 2.5. Water Absorption Characteristics and Kinetics

Adzuki bean samples (3 g) were placed in distilled water (10 mL) and soaked in a water bath at 25 °C for 1, 3, 6, 9, 12, 18, 24, and 30 h [3]. When a specific time was reached, the distilled water was discarded, and the moisture on the surface was removed with the wafer. The weights of the samples were determined before and after soaking and replicated three times. The water absorption behavior of the adzuki beans was calculated using Equation (2). Furthermore, the water absorption kinetics of adzuki beans were investigated by applying Equation (3), which was proposed by Kaptso et al. [17].

(2) $$\mathrm{Water}\;\mathrm{absorption}\;\left(\%,\;d.b\right)=\frac{\mathrm{Soaked}\;\mathrm{sample}\;\mathrm{weight}-\mathrm{Initial}\;\mathrm{sample}\;\mathrm{weight}}{\mathrm{Initial}\;\mathrm{sample}\;\mathrm{weight}}\times 100$$

(3) $$M_{t}=\frac{M_{\mathrm{eq}}}{1+\exp\left[-k\cdot \left(t-\tau \right)\right]}$$

where M_t_ is the moisture content (%, dry basis, d.b.) of the adzuki bean at time t, M_eq_ is the equilibrium moisture content (%, d.b.), τ (h) is the lag phase time (h), and k represents the absorption rate (h^−1^).

### 2.6. Hardness and Softening Kinetics

The hardness of the adzuki bean during soaking was determined according to the method described by Li et al. [18], with a slight modification. Compression testing was performed on a single soaked adzuki bean sample, with 15 replicates per sample, using a texture analyzer (TA-XT plus, Stable Micro Systems Ltd., Godalming, UK). The probe for the compression experiment was a P/5 cylindrical probe (5 mm diameter). The test parameters were as follows: the pre-test and test speeds were set at 5 mm/s; the post-test speed was set at 10 mm/s, and the compression distance was set at 30% strain.

The softening kinetics were calculated using Equation (4) by Miano et al. [9].

(4) $$F_{t}=F_{\infty }+\left(F_{0}-F_{\infty }\right)\cdot e^{-k_{F}\cdot t}$$

where F_0_ is the maximum force to compress at time 0, F_∞_ is the maximum force of penetration at the final stage of cooking (maximum softening), and k_F_ indicates the softening rate.

### 2.7. Extraction

The extraction of control and microwave-treated adzuki beans was performed with a slight modification of the method described by Sung et al. [19]. Briefly, ground samples (1 g) were mixed with 50 mL of distilled water and incubated in a water bath (50 °C) for 3 h. The extracts were filtered through filter paper (Advantec No. 2) and stored at 4 °C before further analysis.

### 2.8. Total Phenolic Compound

The total polyphenol content (TPC) of the sample extract was determined using the Folin–Ciocalteu method [20]. The extract (200 µL) was diluted with 2.6 mL of distilled water, and the Folin–Ciocalteu phenol reagent (200 µL) was added. After reacting for 6 min, 2 mL of Na_2_CO_3_ was added and left at room temperature for 90 min. After 90 min, the absorbance was measured at 750 nm using a spectrophotometer. Gallic acid was used as the standard curve, and the data were expressed as milligrams of gallic acid equivalents per gram (mg GAE)/g) of the dried samples.

### 2.9. Total Flavonoid Compound

Total flavonoid content (TFC) was analyzed by the colorimetric method of Zhishen, et al. [21]. Briefly, 0.25 mL of extract was mixed with 100 µL of distilled water, followed by added 7.5 µL of 5% NaNO_2_. After reacting for 5 min, 15 µL of 10% AlCl_3_ was added and left for another 6 min. After 6 min, 50 µL of 1 M NaOH was added, and the absorbance was measured at 510 nm against the blank. Catechin was used as the standard material, and the TFC was expressed as mg catechin equivalents (CE)/g of the dried sample.

### 2.10. Antioxidant Capacity

The DPPH radical scavenging activity was investigated using a colorimetric method by Kim et al. [22]. Native and microwave-treated adzuki bean extract (20 µL) was added to 200 µL of 0.2 mM DPPH radical solution and allowed to stand for 30 min in the dark. The absorbance at 525 nm was then measured and DPPH radical scavenging activity was represented as mg of vitamin C equivalent (VCE)/g dried sample.

The ABTS radical scavenging activity was measured using the ABTS cation decolorization assay method. A total of 7.4 mM 2,2′-azino-bis (3-ethylbenzothiazoline-6-sulfonic acid) (ABTS, Sigma-Aldrich Co., St. Louis, MO, USA) and 2.6 mM potassium per sulfate were left in the dark for one day to form ABTS cations and diluted with distilled water, so the absorbance value at 735 nm was 1.4. By adding 20 µL of the extract to 200 µL of the diluted ABTS solution, the change in absorbance was measured 30 min later, and the ABTS radical scavenging ability was expressed as VCE/g dried sample.

The ferric reducing antioxidant power (FRAP) was conducted, following the method of Thiapong et al. [23]. Three solutions were prepared: 300 mM sodium acetate buffer (pH 3.6) was prepared using sodium acetate trihydrate and acetic acid, 10 mM TPTZ (2,4,6-tripyridyl-s-triazine) solution in 40 mM HCl, and 20 mM iron chloride hexahydrate. The sodium acetate buffer, TPTZ solution, and iron chloride hexahydrate solution were mixed in a ratio of 10:1:1 and warmed at 37 °C in a water bath before use. The extract (50 µL) was mixed with a FRAP reagent solution (950 µL), followed by standing for 30 min in a dark room. After 30 min, the absorbance was measured at 593 nm, and the results were represented as mg vitamin C equivalent (VCE)/g dried sample.

### 2.11. Statistical Analysis

All experiments were performed at least three times, and the experimental data were expressed as mean ± standard deviation (SD). All experimental data were analyzed using one-way analysis of variance (ANOVA), and Tukey’s test was used to detect significant differences (*p* < 0.05) using SAS software (version 9.4; SAS Institute, Inc., Cary, NC, USA).

## 3. Results and Discussion

### 3.1. Color Analysis

The changes in the color of the control and microwave-treated adzuki beans are shown in Table 1. Color is the fundamental index for customer acceptability; the L, a, and b values of Arari in this study were 40.64, 10.10, and 4.27, respectively. Geomguseul with a black seed coat showed color values lower than those of Arari at 38.07 (L), 0.14 (a), and −0.67 (b). The color of adzuki beans changed gradually and darkened during microwave treatment when examined visually, and the color parameters differed significantly from the control adzuki bean (*p* < 0.05). As the microwave time increased, the lightness and redness of Arari ranged from 37.9 to 40.09 and 5.46 to 8.49, respectively, and the yellowness decreased greatly to 1.13–3.11. A similar tendency was observed in Geomguseul at 60 s (L = 36.78, a = 0.05, and b = −0.87), suggesting darkening, compared to the control. Lightness gradually decreased with increasing heating temperature and time; however, conflicting results have been reported regarding the changes in redness and yellowness. When the sorghum was roasted in a microwave (600 W, 15 min), the lightness sharply decreased. However, the redness and yellowness were increased from 1.75 to 7.66 and 12.7 to 21.2, respectively [24]. The heating process, such as roasting, affected the color of the extracts, and Song et al. [25] reported that the roasted adzuki bean extracts for developing products showed a decrease of lightness and increase of both redness and yellowness. The color difference (ΔE) after microwave treatment showed a significant change, especially in the ΔE of Arari (2.51–6.23), which showed a change greater than Geomguseul (0.64–1.54). The color changes (L, a, b, and ΔE) were greater without a resting time, even if the microwave treatment time was the same. Therefore, it is necessary to select an appropriate treatment time (with or without resting time), with consideration of the color change during the microwave treatment of adzuki bean.

The color changes were associated with Maillard reactions and caramelization during heating, and Krysiak et al. [26] suggested the physical and chemical changes of the bean, depending on the temperature, as follows: (1) tissue destruction and structural changes, including protein denaturation at 50–60 °C; (2) non-enzymatic browning and water evaporation of bean seed over at 100 °C; (3) thermal decomposition into volatile compounds, as well as caramelization, oxidation, and esterification above 180 °C. Yao et al. [27] suggested that the overall color of the adzuki beans showed a sharp decline, due to the thermal accumulation inside the baked beans resulting from microwave heating (*p* < 0.05), which caused an obvious Maillard reaction in the adzuki beans in a short time.

### 3.2. Microstructure

**Figure 2:**
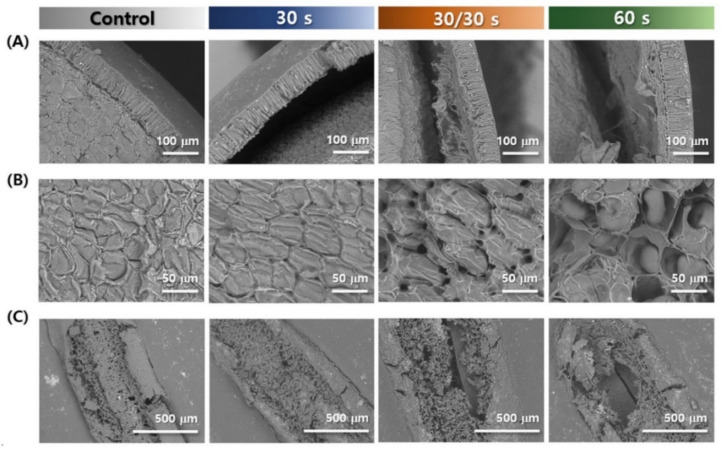
Microstructure characteristics of Arari (red-colored adzuki bean), depending on microwave treatment; seed coat (**A**), cotyledon (**B**), and hilum (**C**).

**Figure 3:**
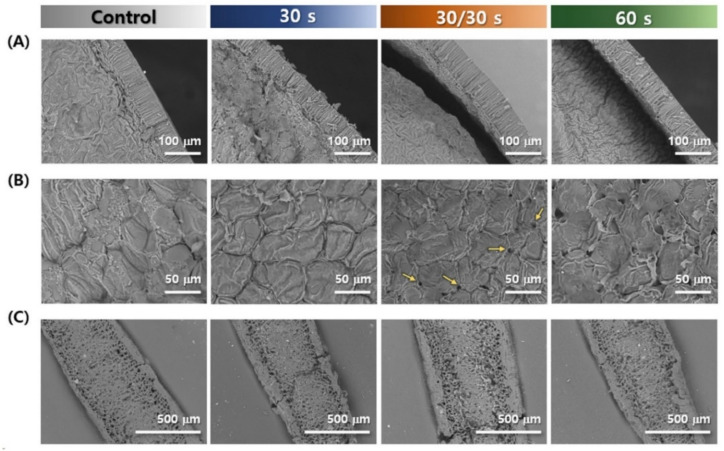
Microstructure characteristics of Geomguseul (black-colored adzuki bean), depending on microwave treatment; seed coat (**A**), cotyledon (**B**), and hilum (**C**).

The seed coat, cotyledon, and hilum of the two adzuki bean varieties, depending on the microwave treatment, are shown in Figure 2 and Figure 3. As shown in Figure 2A and Figure 3A, it was confirmed that the hilum of the adzuki bean had cells arranged in a porous honeycomb or crosswise arrangement, similar to other beans [28,29], whereas the control group and 30 s treated Arari still had the hilum covered with a dense structure; the evidence shows that part of the hilum was damaged in the cases of 30/30 and 60 s. Meanwhile, the hilum of the microwave-treated Geomguseul was not greatly different from that of the control, and there was no damage in it. Many studies have suggested that the hilum is an important region for determining the water absorption quantity and rate, and Miano et al. [30] found that water absorption was first routed from the hilum and hilar fissure and then distributed to the seed coat and the cotyledon via the diffusivity of the colorant. Furthermore, the water absorption kinetics of hilum- and microphyll-covered white kidney beans were significantly reduced, compared to those of normal grains [31]. Therefore, this damage to the hilum would be an effective way to improve the water absorption of grains.

The seed coats of legumes were divided into three parts: cuticle, palisade, and osteosclereid cells (hourglass cells) [8]; however, a slight structural difference depends on the legumes, which affects the mass transfer of water. Unlike other beans, such as soybean, black bean, and cowpea [32], hourglass cells were not found in the adzuki beans, which is consistent with this study (Figure 2B and Figure 3B). Organized and elongated palisade cells were densely arranged, and a multilayer of parenchyma cells was horizontally present, without hourglass cells. The average thicknesses of Arari and Geomguseul were 58.3 and 56.6 μm, respectively, which is consistent with a previous study that reported that the thickness of seed coat was distributed from 40 to 60 μm [3,10]. It was found that the space between the seed coat and cotyledon as microwave treatment time was longer because the water evaporation led to break down of the cell membrane and layer. Miano and Augusto [33] suggested that the microscopic space between the seed coat and cotyledon induces a slow hydration process. Therefore, it is expected that the formation of a larger space and cell rupture by the microwave treatment can contribute to the free distribution of water.

Figure 2C and Figure 3C represent the cross-section of the cotyledon from the control and microwave-treated Arari and Geomguseul, respectively. The cotyledons of the two control beans were adjacent to the parenchyma cell layer of the seed coat, and the storage cells, arranged closely inside the cotyledon, facilitated hydration in the cotyledon via capillaries in the intercellular space. Engquist and Swanson [10] reported that the density of intercellular storage cells in adzuki beans varied, depending on the cultivar. As seen in the morphology of cotyledon in 30/30 s and 60 s, it could be identified that microwave heating affected the structure of cotyledon; especially, there were greater changes in Arari than Geomguseul. Microwave-treated rapeseed and camellia seeds revealed a denaturation of the proteins and lipids, with evident porosity and many irregular cavities, due to the destruction of cell structure [34,35]. In this study, even if the exposure to microwave treatment time was the same, continuous treatment proceeded much more toward cell destruction. Therefore, starch embedded in the protein matrix in the storage cell was distinct, as shown in 60 s Arari.

### 3.3. Water Absorption Behavior and Kinetics

**Figure 4:**
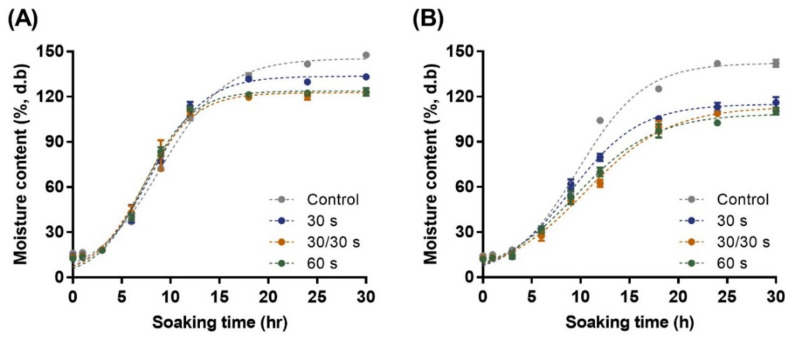
Water absorption behavior of two adzuki bean varieties with microwave-treatment during soaking (25 °C): Arari (**A**) and Geomguseul (**B**). The error bars indicate the standard deviation, and the dotted lines in the figure were from the softening behavior obtained from Equation (3).

In legumes, water absorption behavior is regarded as an important factor that affects germination, cooking time, quality, texture, and sensory characteristics. The hydration behavior of beans appears in two types: downward concave shape (DCS) and sigmoidal shape behaviors. The DCS behavior shows rapid hydration soon after soaking and reaches equilibrium, whereas the sigmoidal behavior features slow hydration (lag phase) during the initial soaking period, followed by a rapid increase and plateau. As shown in Figure 4, the adzuki beans exhibited sigmoidal water absorption behavior, regardless of the variety and microwave treatment. In addition, it takes approximately 2.5–3 h to terminate the lag phase in both Arari and Geomguseul. The appearance of the lag phase is due to the impermeability of the seed coat, and it is the period it takes to reach a specific water activity to convert the glassy state of the seed coat to a rubbery state [33,36]. This time varies depending on various factors, such as the soaking temperature [37], varieties [3,36], and initial moisture content of beans [33]. After the lag phase, the resistance by the impermeability of the seed coat is decreased, and mass flow increased into the grain surface and hilum, until the inflection points with fast water absorption kinetics. However, the water absorption kinetics are reduced when the maximum water absorption level is reached because the driving force decreases after the inflection point [38].

The control and microwave-treated Arari showed an increased tendency within 12 h, after which, a notable characteristic of equilibrium moisture content was observed. As the microwave treatment was extended, equilibrium moisture content (M_eq_) was reduced, and control had a M_eq_ of 145.3%, whereas 30 s Arari had 133.6%, followed by 122.7–123.8% at 30/30 s and 60 s. This result is also clearly identified in Geomguseul, especially after 6 h. The microwave-treated samples showed a gradual increase in water absorption, compared to the control, and the equilibrium moisture content was over 140% for the control; however, the microwave-treated samples were less than 115%. The equilibrium moisture content is defined as the moisture content when the water vapor pressure present in food is in equilibrium with its environmental surroundings. Concerning M_eq_, seeds with an initial water content that is too low (4.23%) showed a low M_eq_, which is only 20% of the M_eq_ of the seed with 15% initial water content [33]; the M_eq_ of white kidney bean [30], adzuki bean [36], and Andean lupin [30] changed depending on the soaking temperature. The decrease in M_eq_ is attributed to the higher extraction of the water-soluble components of the grain at higher temperatures [39]. Oliveira et al. [37] proposed that higher temperatures damage cell membranes, resulting in lower water holding capacity. Another possibility is that, as the hydration rate is faster at higher temperatures, the seed edges quickly absorb water, which reduces the mass transfer from the soaking water to the grain surface when the external layer is saturated.

From the many studies, theoretical and empirical formulations, such as the Peleg’s [40], Ibarz-Augusto [41], and Weibull [42] models, were proposed to elucidate the water absorption kinetics. Generally, Peleg’s model has been widely used to calculate the hydration kinetics of grains, such as wheat, maize, and sorghum [9]. However, it is not adequate for sigmoidal behavior with a lag phase in initial soaking [34,38]. Piergiovanni [43] investigated the hydration kinetics of various common bean cultivars and showed that Peleg’s model was not always suitable for determining the hydration kinetics. Kaptso et al. [17] proposed a kinetic hydration model with three parameters (M_eq_, τ, and k) considering the lag phase, and Oliveira et al. [37] suggested that it is suitable to describe the hydration kinetics of adzuki beans. Therefore, the water absorption kinetics in our study were obtained using the model equation from Kaptso et al. [17], which demonstrated a good fit, as R^2^ ranged from 0.986 to 0.994 (Table 2). The initial moisture contents (M_0_) of Arari and Geomguseul were 16.17 and 14.40%, respectively; however, the moisture content decreased to approximately 12%, owing to evaporation by microwave treatment. In Arari, the inflection times (τ) were decreased from 9.01 to 7.24 h; however, the inflection point of the Geomguseul had no tendency, as 9.72 h for the control, and 9.03 h, 10.46 h, and 9.48 h for microwave-treated samples (30 s, 30/30 s, and 60 s), respectively. The water absorption kinetics of Arari was 0.30 h^−1^, whereas, the kinetics was increased after microwave treatment from 0.36–0.41 h^−1^. However, microwave-treated Geomguseul decreased the water absorption rate, compared to the control, except for the sample treated for 30 s. Therefore, it is suggested that microwave treatment promotes the inflow of water and improves the water absorption rate in Arari, which corresponds to the inflection point results. The different aspects of the two adzuki bean varieties may be attributed to their components, microstructures, and susceptibility to microwaves. Microwave treatment could form an appropriate space for water to flow widely inside the adzuki bean; however, it has been proposed that some proteins or lipids may cause hardening, which becomes vulnerable to moisture absorption when cooled after microwave treatment. Thus, it is necessary to set the appropriate microwave treatment conditions to control the water absorption rate of adzuki beans, depending on the processing purpose.

### 3.4. Softening Behavior and Kinetics

**Figure 5:**
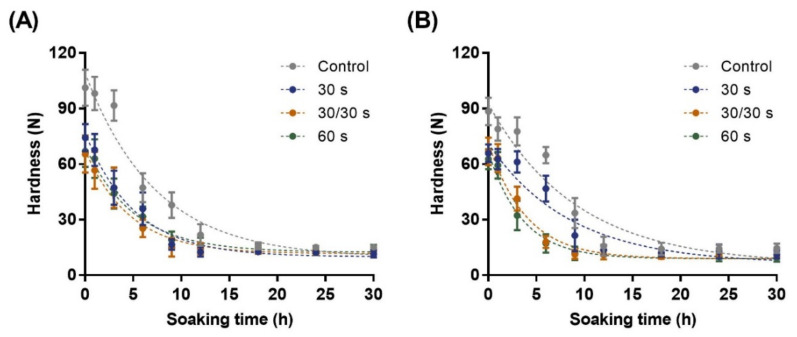
Softening behavior of two adzuki bean varieties with microwave-treatment during soaking (25 °C): Arari (**A**) and Geomguseul (**B**). The error bars indicate the standard deviation, and the dotted lines in the figure were from the softening behavior obtained from Equation (4).

The softening behavior of beans during soaking is considered one factor that predicts processing and cooking quality. Some studies have classified the change in the hardness of soaked soybeans into two or three phases. During soaking at room temperature, the lentil showed three phases: initial slow softening within approximately 60 min (1), rapid softening during 60–150 min (2), and a constant phase after 150 min (3) [44]. With chickpea [45], there were two phases: rapid softening until 4 h, and subsequent gradual softening, which approached the minimum hardness from 4 to 16 h. In this work, Figure 5 shows the softening behavior of adzuki beans with or without microwave treatment during soaking, which can be divided into two or three phases, depending on the sample. Softening behavior composed of three phases was found in both control adzuki beans, as well as 30 s Geomguseul, and other microwave-treated adzuki beans exhibited the softening behavior of the two phases.

Control Arari and Geomguseul of initial time had a hardness of 101.2 and 88.4 N, respectively, due to their hard shell. However, the two microwave-treated adzuki bean varieties showed a 30–35% decrease in hardness, compared to the initial hardness, ranging from 62.2–75.6 N. between the seed coat-cotyledon and cavities within cotyledon, resulting in fragility to compress force. Absorbed microwaves are converted into thermal energy, which breaks down tissue in the cell, resulting in increased brittleness [34,35]. In the control of both adzuki bean varieties, the hardness decreased slowly for up to 3 h, corresponding to the water absorption behavior. Thus, it could be regarded as the hardness of the adzuki bean in a glassy state because a sufficient amount of water in the seed coat was neither absorbed nor uniformly distributed during the initial soaking period. In contrast, microwave-treated adzuki beans, except for 30 s Geomguseul, showed a significant decrease in hardness, until 9 h for Arari and 6 h for Geomguseul, when they were immersed. Although the minimum hardness was less than 20 N, which was not significantly different among the samples; the time taken to enter the equilibrium hardness phase was affected by the presence or absence of microwave treatment. In the case of the control group, over 15 h were required, whereas those of microwave-treated Arari and Geomguseul were shortened to 12 and 9 h, respectively. As shown in Table 3, the hardness softening rate of the adzuki bean was 0.1369 h^−1^ for the control group, and the rate increased to 0.2142 h^−1^ with microwave treatment. The softening rate of Geomguseul, which was 0.1091 for the control, increased to 0.1180 (30 s), 0.2561 (30/30 s), and 0.2835 (60 s). The influx of water into the adzuki bean and its softening proceed actively, owing to the structural deformation caused by microwave treatment; however, the irregular structure caused by heat denaturation interferes with the constant water distribution between the cells of the cotyledon, resulting in a slow softening rate in some samples.

### 3.5. TPC, TFC, and Antioxidant Activities

The bioactive content of adzuki bean is high; in particular, the content of TPC and TFC in the seed coat is high enough to account for over 90% of the adzuki bean seed [46]. Furthermore, the color of the seed coat was considered a factor for determining the concentration of phenolic compounds, flavonol glycosides, anthocyanins, and condensed tannins. Representatively, proanthocyanidin has high antioxidant activity and antibacterial, antiviral, anticancer, anti-inflammatory, antiallergic, and vasodilatory properties. Thus, this study aimed to determine how adzuki beans with different seed coat colors are affected by microwaves.

**Figure 6:**
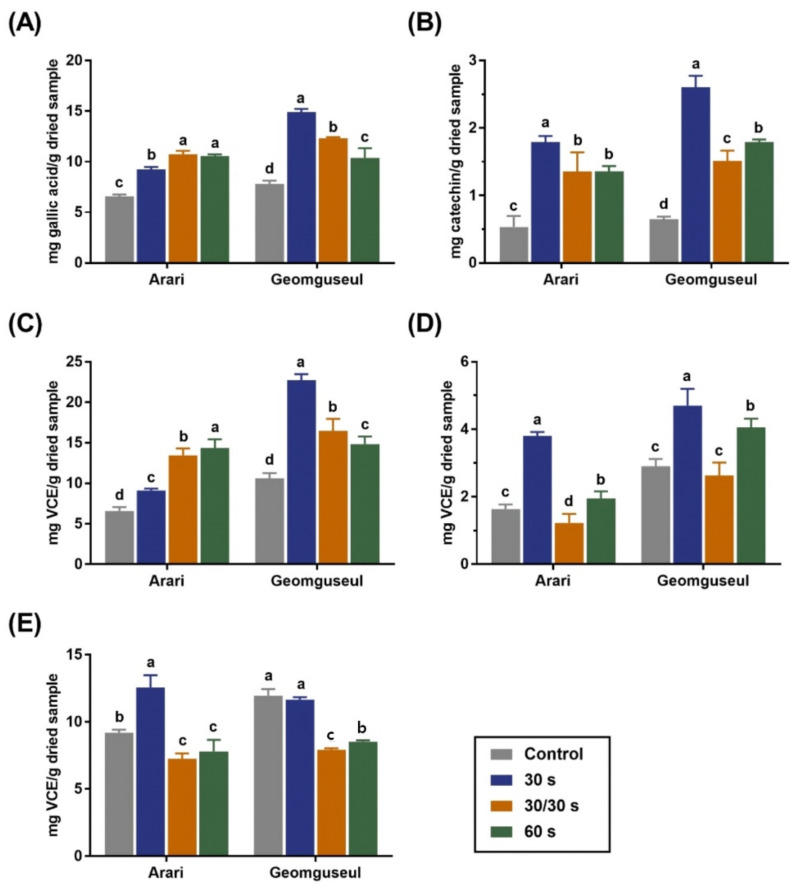
Total phenolic content (**A**), total flavonoid content (**B**), ABTS radical scavenging capacity (**C**), DPPH radical scavenging capacity (**D**), and FRAP assay (**E**) of two adzuki bean varieties, depending on microwave treatment. The same superscript letters within the same cultivar indicate no significant differences (*p* < 0.05).

The TPC, TFC, and antioxidant activities of the adzuki bean with and without microwave treatment are shown in Figure 6. The TPCs (Figure 6A) of the control Arari and Geomguseul dried samples were 6.57 and 7.81 mg GA/g, respectively, which were higher than the results obtained when extracted with 80% fermented alcohol [25]. According to Lee et al. [47], adzuki beans had a higher polyphenol compound content than black beans, which contradicts our study. Two adzuki bean varieties with different seed colors showed different TPC changes after microwave treatment. With Arari, the TPC increased as the microwave treatment time increased, whereas with Geomguseul, the microwave treatment for 30 s showed the highest TPC content, which decreased over time. The TFCs (Figure 6B) of control adzuki beans with less than 1.00 mg CE/g of the dried sample increased to 1.79 mg CE/g of the dried sample for the microwave-treated Arari and 2.60 mg CE/g dried sample for microwave-treated Geomguseul, respectively. In particular, microwave treatment for 30 s was suitable for maximizing flavonoid content; however, longer microwave treatment significantly reduced flavonoid content (*p* < 0.05). The increase in TPC and TFC is thought to be due to the generation of aminocarbonyl reaction products, such as melanoidin, which have a high free radical scavenging ability because of heat generated by microwaves. Yao et al. [27] reported that the heating process leads to precursors of volatile substances, and the thermal decomposition of oligosaccharides generates reducing sugars. Consequently, a reaction between the free amino acids and reducing sugars releases furans, pyrazines, etc. However, flavonoid and phenolic components were susceptible to thermal processing; thus, excess thermal processing resulted in a decrease in TPC and TFC.

Phenolic compounds are regarded as the major compounds that contribute to the total antioxidant activity of grains. In ABTS radical scavenging activity (Figure 6C), Arari and Geomguseul showed 6.57 mg and 10.61 mg VCE/g of the dried sample, respectively, whereas it is obtained 1.63 mg VCE/g of the dried sample for Arari and 2.91 mg VCE/g of the dried sample in DPPH radical scavenging (Figure 6D). ABTS produces cationic radicals and can measure both lipophilic and hydrophilic substances, whereas DPPH produces anionic radicals and represents the antioxidant capacity of lipophilic substances. Therefore, these two methods may have different measurement results because of the different degrees of bonding between the substrate and the reactant. With Arari, ABTS at 30/30 s and 60 s was higher than the control group, whereas with DPPH, it was lower. From this, the evidence shows that an increase in microwave time reduces the antioxidant activity of the fat-soluble components. With Geomguseul, excellent ABTS and DPPH radical scavenging abilities were obtained when microwave treatment was performed for 30 s, as in the TPC and TFC results. However, as the microwave treatment time increased, both radical scavenging activities decreased. The FRAP results (Figure 6E) show that the control Arari was 9.19 mg VCE/g of the dried sample, and it increased significantly to 12.55 mg VCE/g sample after 30 s of microwave treatment. However, a smaller amount was detected, compared to the control (7.25–7.78 mg VCE/g dried sample), as the microwave treatment time increased. Furthermore, there was no significant difference between the control and 30 s Geomguseul; similar to Arari, the value that was over 11.0 mg VCE/g of the dried sample was reduced to 7.9–8.5 mg VCE/g of the dried sample. Ashraf et al. [48] reported there was a significant difference between the native and heat-treated peptides isolated from adzuki bean. The FRAP results were more similar in tendency to the DPPH results, compared to than ABTS, which is in agreement with a previous study [49]. Different antioxidant properties were reported according to the heat treatment time; the antioxidant activities decreased after 15 min of microwave treatment and increased after 30 min of treatment, depending on the type of cowpea [50]. In addition, it differs depending on the heat treatment method. Compared with microwave treatment for the same duration, autoclave treatment resulted in a decrease in antioxidant activity.

## 4. Conclusions

The novelty of this study is inducing a changes of characteristics through the short-time microwave treatment of dried adzuki bean seeds. Microwave treatment was applied to change processing-related properties, including the color, microstructure, water absorption, softening, and functional properties of Korean adzuki beans. Regardless of seed coat color, the lightness, redness, and yellowness decreased as the microwave treatment time increased, due to the Maillard reaction. Microwave treatment affected the space formed between the seed coat and cotyledon of adzuki beans, as well as the formation of cavities, by removing water from the storage cells. Although the changes in the internal structure of adzuki beans varied, depending on the cultivar, microwave treatment increased both the rate of water absorption and softening in hardness, suggesting that the problem of the long soaking times of adzuki beans before further processing could be improved with microwave pretreatment. Moreover, microwave-treated adzuki beans showed a higher TPC, TFC, and antioxidant capacity than the untreated adzuki beans; however, excessive microwave treatment time decreased the bioactive compound. Considering industrial application, microwave heating is a method with a short processing time (<5 min) that is energy-saving and high-efficiency, compared to conventional heat treatment. Therefore, it is expected to be actively applied as a pretreatment method for legumes, such as red beans, in order to improve water absorption, softening, and antioxidant activities, based on the result of this study.

## Figures and Tables

**Table 1:** Color changes in two varieties of adzuki beans, depending on microwave treatment.

		L (Lightness)	a (Redness)	b (Yellowness)	ΔE
Arari	Control	40.64 ± 0.23 ^a^	10.10 ± 0.29 ^a^	4.27 ± 0.16 ^a^	-
	30 s	39.09 ± 0.36 ^b^	8.49 ± 0.35 ^b^	3.13 ± 0.31 ^b^	2.51 ± 0.18 ^c^
	30/30 s	38.62 ± 0.21 ^c^	6.79 ± 0.38 ^c^	1.91 ± 0.24 ^c^	4.54 ± 0.10 ^b^
	60 s	37.90 ± 0.25 ^d^	5.46 ± 0.24 ^d^	1.13 ± 0.14 ^d^	6.23 ± 0.04 ^a^
Geomguseul	Control	38.07 ± 0.12 ^a^	0.14 ± 0.05 ^a^	−0.67 ± 0.02 ^a^	-
	30 s	37.51 ± 0.51 ^b^	0.08 ± 0.03 ^ab^	−0.63 ± 0.13 ^a^	0.64 ± 0.10 ^b^
	30/30 s	37.15 ± 0.54 ^bc^	0.09 ± 0.08 ^ab^	−0.87 ± 0.14 ^b^	1.17 ± 0.22 ^a^
	60 s	36.78 ± 0.37 ^c^	0.05 ± 0.01 ^b^	−0.87 ± 0.07 ^b^	1.54 ± 0.22 ^a^

The same superscript letters within the column of the same cultivar indicate no significant differences (*p* < 0.05).

**Table 2:** Water absorption parameter and kinetics of microwave-treated adzuki beans using the sigmoidal model with permission from Kaptso et al [16]. (2022, Kaptso, K.G.).

Sample		M_0_ (%, d.b.)	M_eq_ (%)	τ (h)	k (h^−1^)	R^2^
Arari	Control	16.17 ± 0.06 ^a^	145.3 ± 1.74 ^a^	9.01 ± 0.18 ^a^	0.30 ± 0.01 ^c^	0.994
	30 s	14.15 ± 0.12 ^b^	133.6 ± 1.58 ^b^	7.89 ± 0.17 ^b^	0.36 ± 0.02 ^b^	0.993
	30/30 s	13.54 ± 0.06 ^c^	122.7 ± 1.67 ^c^	7.24 ± 0.38 ^b^	0.38 ± 0.02 ^b^	0.990
	60 s	12.56 ± 0.13 ^c^	123.8 ± 1.41 ^c^	7.32 ± 0.15 ^b^	0.41 ± 0.03 ^a^	0.993
Geomguseul	Control	14.40 ± 0.07 ^a^	142.3 ± 2.48 ^a^	9.72 ± 0.28 ^b^	0.29 ± 0.02 ^b^	0.986
	30 s	12.65 ± 0.11 ^b^	114.0 ± 1.53 ^b^	9.03 ± 0.21 ^c^	0.31 ± 0.01 ^a^	0.993
	30/30 s	12.77 ± 0.03 ^b^	113.3 ± 1.80 ^b^	10.46 ± 0.27 ^a^	0.23 ± 0.03 ^c^	0.992
	60 s	12.04 ± 0.12 ^b^	108.6 ± 1.48 ^b^	9.48 ± 0.23 ^bc^	0.25 ± 0.01 ^c^	0.993

The same superscript letters within the column of the same cultivar indicate no significant differences (*p* < 0.05) using Tukey’s test. The water absorption and kinetic parameter (M_eq_, τ, and k) were obtained from Equation (3) and are represented as mean ± standard error.

**Table 3:** Softening kinetics of microwave-treated adzuki beans using the model with permission from Miano, Sabadoti, and Augusto et al [9]. (2022, Miano, A.C.).

Sample		Softening Equation	k (h^−1^)	R^2^
Arari	Control	$F_{t}=9.44+\left(109.2-9.44\right)\cdot e^{0.1369\cdot t}$	0.1369 ± 0.0322	0.9613
	30 s	$F_{t}=9.09+\left(76.81-9.09\right)\cdot e^{0.1940\cdot t}$	0.1940 ± 0.0275	0.9836
	30/30 s	$F_{t}=11.62+\left(67.40-11.62\right)\cdot e^{0.2142\cdot t}$	0.2142 ± 0.0346	0.9786
	60 s	$F_{t}=12.42+\left(69.68-12.42\right)\cdot e^{0.2076\cdot t}$	0.2076 ± 0.0218	0.9899
Geomguseul	Control	$F_{t}=5.79+\left(92.57-5.79\right)\cdot e^{0.1091\cdot t}$	0.1091 ± 0.0381	0.9305
	30 s	$F_{t}=6.10+\left(71.13-6.10\right)\cdot e^{0.1180\cdot t}$	0.1180 ± 0.0400	0.9301
	30/30 s	$F_{t}=8.88+\left(71.57-8.88\right)\cdot e^{0.2561\cdot t}$	0.2561 ± 0.0445	0.9752
	60 s	$F_{t}=8.90+\left(66.72-8.90\right)\cdot e^{0.2835\cdot t}$	0.2835 ± 0.0444	0.9798

The softening parameter (*k*) was obtained from Equation (4) and was represented as mean ± standard error.

## Data Availability

The data presented in this study are available in the article.
